# Antibody-oligonucleotide conjugates: an emerging modality for precision RNA therapeutics

**DOI:** 10.1093/abt/tbag022

**Published:** 2026-04-29

**Authors:** Chen-Hsu Yu, Summer Y Y Ha, Zhiqiang An, Kyoji Tsuchikama, Wenbo Li

**Affiliations:** Department of Biochemistry and Molecular Biology, McGovern Medical School, University of Texas Health Science Center at Houston, Houston, TX 77030, United States; Texas Therapeutics Institute, The Brown Foundation Institute of Molecular Medicine, McGovern Medical School, The University of Texas Health Science Center at Houston, Houston, TX 77030, United States; Texas Therapeutics Institute, The Brown Foundation Institute of Molecular Medicine, McGovern Medical School, The University of Texas Health Science Center at Houston, Houston, TX 77030, United States; Texas Therapeutics Institute, The Brown Foundation Institute of Molecular Medicine, McGovern Medical School, The University of Texas Health Science Center at Houston, Houston, TX 77030, United States; Department of Biochemistry and Molecular Biology, McGovern Medical School, University of Texas Health Science Center at Houston, Houston, TX 77030, United States; Texas Therapeutics Institute, The Brown Foundation Institute of Molecular Medicine, McGovern Medical School, The University of Texas Health Science Center at Houston, Houston, TX 77030, United States

**Keywords:** RNA therapy, antibody-oligonucleotide conjugate (AOC), genetic diseases, multi-specific drugs

## Abstract

RNA-targeting therapeutics have enormous potential to precisely target disease-causing RNAs, extending beyond the traditional limits of “druggability” for small molecules, antibodies, and protein-targeting cell therapies. However, one crucial limitation is that RNA-targeting drug modalities (such as oligonucleotides) cannot effectively reach diseased tissue or cell types. Antibody-oligonucleotide conjugates (AOCs) emerge as a promising frontier in aiding RNA therapeutics by harnessing antibodies to deliver drug modalities to target specific RNAs in desired tissues or cells. In this Review, we summarize the critical components of AOCs, key considerations for their design and manufacturing, ongoing AOCs in preclinical/clinical development, and disease indications. We discuss the current hurdles to improving AOC efficacy and extending its application, concluding with an outlook on the unique opportunities offered by AOCs beyond traditional oligonucleotides and small-molecule antibody-drug conjugates. We propose that, with focused efforts to overcome key challenges, AOCs have the potential to transform RNA therapeutics, offering treatment options for many previously untreatable diseases.

Statement of Significance We reviewed the design principles of antibody-oligonucleotide conjugates and shared our perspectives on the challenges and opportunities to realize the potential of this emerging drug modality to treat various human diseases.

## Introduction

**Figure 1:**
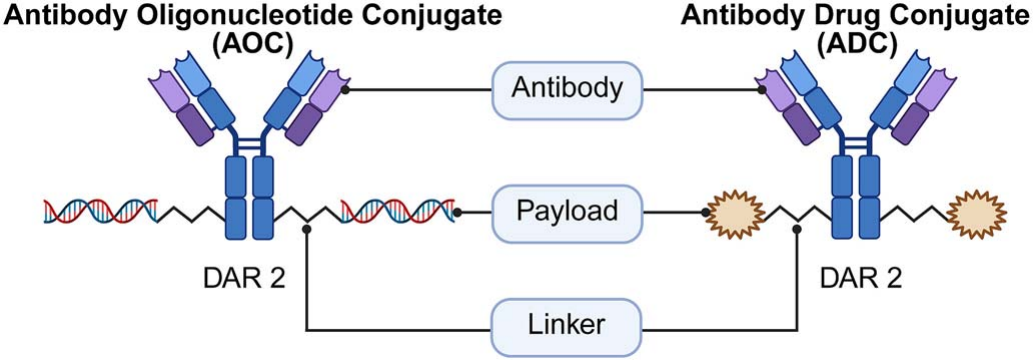
Schematic illustration of an AOC and ADC, both consisting of an antibody, linker, and payload; while they share similar structural frameworks, the major distinction lies in their payloads—hydrophobic small-molecule drugs in ADCs versus highly charged nucleic acid sequences in AOCs, which leads to a series of physicochemical differences in conjugation strategies, linker chemistry, stability, manufacturing, and pharmacology; for this review, the term ADC refers specifically to antibodies conjugated to small-molecule drug payloads; the diagram shows that the example of AOC or ADC bears a drug-to-antibody ratio (DAR) of 2; DAR is an important feature for AOC or ADC drugs that impact the drugs’ various properties and efficacy (Created in BioRender).

Monoclonal antibodies (mAbs) represent a major drug modality that has revolutionized the treatment of various diseases, particularly in oncology and autoimmune disorders [1–4]. By binding to circulating or cell-surface antigens, they can either neutralize/modulate cellular signaling or facilitate drug delivery. Besides the early success of neutralizing antibody drugs, such as trastuzumab, recent prominent applications of mAbs include immune-checkpoint blockade immunotherapy [5] and antibody-drug conjugates (ADCs) in oncology [6, 7] (Fig. 1). These therapeutics showed excellent efficacy and high precision (minimal off-target effects). The success of ADCs inspired studies to use antibodies to deliver other drug modalities.

RNA medicine has made significant progress [8], propelled by a better understanding of the human transcriptomes [9] and the development of effective modalities to manipulate RNAs [10]. Oligonucleotides (ONs) are currently the dominant RNA-targeting modalities, exemplified by small interfering RNAs (siRNAs), antisense oligonucleotides (ASOs), phosphorodiamidate morpholino oligomers (PMOs), and aptamers. Since 2016, 8 siRNA and 14 ASO drugs have been approved by the FDA for the treatment of genetic disorders, cancers, and neurological diseases [10–12]. Many of these diseases are otherwise untreatable. ON drugs are appealing because they can potentially target two-thirds of the human genome that produces RNAs, as compared to only about 2% that makes proteins [9], not to mention that only ~20% of proteins are considered druggable [13, 14].

However, ON drugs still face critical challenges in clinical application, including limited stability and rapid clearance from circulation, difficulties in crossing biological barriers such as the blood–brain barrier (BBB) and cell membranes, and preferential retention in specific tissues, particularly the liver, if administered systemically [15, 16] (Table 1). As such, approved siRNA drugs are most used to treat hepatic diseases [17]. For ASOs, while they show promise for neurological diseases, they need to be administered via intrathecal delivery, which remains diffusion-mediated, invasive, and challenging to operate [10, 15].

**Table 1:** Comparative summary of the relative strengths and limitations of AOCs, unconjugated ONs, and ADCs.

Modalities	Strengths	Limitations
AOCs	Combines two specificities: from antibody and RNA targeting, respectively Antibody-mediated cell- and tissue-specific delivery of ONs Extended circulation half-life compared with free ONs Modular design allows tunable DAR, linker chemistry, and payload types Reduce systemic off-target exposure	Inefficient endosomal escape remains a major intracellular barrier DAR-dependent effects on antibody binding, PK, and stability Hybrid protein–polyanion nature creates analytical and formulation challenges Risk of aggregation and altered biodistribution due to ON charge Limited clinical experience and immature regulatory precedent compared to ADC
ONs	Sequence-specific modulation of gene expression (RNAi, RNase H, exon skipping) Simple and scalable synthesis with well-established chemical modifications Multiple clinically approved therapeutics across diverse indications Extensive chemical modification toolbox to enhance stability and potency	Endosomal trapping severely limits effective intracellular delivery Rapid renal and hepatic clearance, resulting in a short plasma half-life Poor cellular and tissue specificity Often require high doses or frequent administration Dependence on invasive routes if via extrahepatic delivery Off-target accumulation in the liver and kidney
ADCs	Clinically validated platforms, tremendous success in oncology; growing exploration in infectious, autoimmune, and inflammatory diseases High target specificity through antibody-mediated delivery Highly effective for oncology via potent cytotoxic payloads Mature linker chemistry and site-specific conjugation strategies Established cGMP manufacturing and regulatory frameworks	Payloads are typically cytotoxic and not suitable for gene modulation Hydrophobic payloads and high DAR can cause aggregation and rapid clearance Limited to protein-level targeting, not gene regulation, constraining therapeutic scope Risk of off-target toxicity due to premature release of cytotoxic payloads or bystander effect to unintended tissues expressing the antigen Development of resistance mechanisms (antigen loss, efflux pumps)

Antibodies can facilitate ON delivery, catalyzing the development of a field of antibody-oligonucleotide conjugates (AOCs) [18–20] (Fig. 1). Early efforts in the 1990s already attempted to conjugate ONs with antibodies for better cellular delivery [21, 22]. Encouraging progress was achieved recently, with the first clinical trial of AOC taking place since 2021 [19], which is projected to reach the market by 2027 [23]. By linking ONs to mAbs, AOCs possess their respective strengths while avoiding their weaknesses (Table 1). Indeed, AOCs enjoy the tissue- and cell-targeting specificity and extended circulation time conferred by mAbs, which overcome the limitations of ONs. AOCs still retain the broad targeting ability for virtually any RNA molecule conferred by ONs, overcoming the limitations of small-molecular payloads in current ADCs. These include mRNAs for traditionally “undruggable” proteins [e.g. transcription factors (TFs) such as *MYC* [24]], driver genes that frequently mutate and become resistant to small-molecule drugs [e.g. Kirsten rat sarcoma viral oncogene homolog (KRAS) [25]], mRNA splicing isoforms (e.g. Survival of motor neuron 2 (*SMN2*) [26]), and emerging disease-associated noncoding RNAs [27] (Table 1). These strengths make AOCs a therapeutic approach with potentially unprecedented effectiveness, precision, and limitless targets.

However, it is noteworthy that AOCs are compositionally diverse molecules. The two distinctive categories of molecules, antibodies and ONs, both possess substantial variabilities due to different antigens, epitopes, antibody characteristics (for antibodies), and ON classes, action compartments in a cell, and modifications (for ON). Their variable physicochemical properties require tailored, chemically-diverse linkers to ensure AOC efficacy, pharmacokinetics (PK)/pharmacodynamics (PD), and developability. The complexity of each of the three components of AOCs incurs challenges that need to be addressed.

Herein, we first provide an overview of general considerations for AOCs, including antigen/antibody selection and development, ON types and features, target RNAs, conjugation methods and linker chemistry, AOC mechanisms of action, tissue and cellular fates, and disease indications under development. We elaborate on current opportunities and critical challenges, providing our perspective on how to realize the transformative potential of AOCs in treating various human diseases.

## Design and engineering of antibody-oligonucleotide conjugate

### Antigen selection and antibody development

An ideal antigen for AOC should be specifically and/or highly expressed in diseased cells, but less so by irrelevant cells, thereby minimizing off-target effects. In cancer, tumor-specific or overexpressed antigens, such as human epidermal growth factor receptor 2 (HER2), have been successfully explored for the development of antibody therapeutics and ADCs [28, 29]. In neurological disease, the transferrin receptor-1 (TfR1) is a commonly used antigen because it can facilitate antibodies to cross the BBB into the central nervous system (CNS) [30–33]. TfR1 is also well expressed in muscle cells, allowing AOC development for neuromuscular diseases [34]. Bispecific antibodies, designed to recognize a disease-specific antigen and a transport receptor, can also be employed to develop AOCs [35, 36].

Antibody engineering critically considers epitopes, immunotypes, and lengths/formats. First, epitopes affect the affinity and specificity of antibodies toward the antigen. It is crucial to select the epitope of an antigen in its native, folded conformation so that the therapeutic antibody can recognize the antigen in living cells and tissues [4]. Second, antibody species and immunotypes can impact the characteristics and immunogenicity of antibody drugs [37]. For example, IgG is commonly used in current ADCs due to longer half-life and effective engagement of immune effectors [37]. Immunogenicity of antibody drugs can cause immune problems in patients, including the production of anti-drug antibodies (ADAs), which can be mitigated by antibody humanization [38]. Third, different antibody formats can be chosen for tailored purposes. Full-length mAbs have more sites for drug conjugation and are more stable; however, their tissue penetration can be less optimal [39]. For AOCs, site-specific conjugation of ONs to antibodies depends on specific amino acids that may only exist in full-length [40]. Antibody fragments, including fragment antigen-binding (Fab), single-chain fragment variable (scFv), and other smaller antibody types such as nanobodies, offer better penetration but may suffer from reduced stability in circulation [39]. Their shorter length also limits their utility for site-specific conjugation. As a result of these considerations, mAb engineering employs affinity maturation and humanization to reduce immunogenicity, optimize binding kinetics, and improve PK [41]. Antibodies can also be subjected to chemical modifications such as PEGylation, to improve biodistribution and half-life in circulation [42, 43].

Antigen/antibody properties strongly influence the efficacy and pharmacology of ON payloads. One study conjugated siRNAs to seven different antigen/antibody pairs, finding that only two pairs showed significant target mRNA knockdown [44]. Interestingly, even the anti-HER2 antibody commonly used in ADCs did not show a clear knockdown in this study. However, it is noteworthy that other reports have found good efficacy of anti-HER2 AOCs in reducing target RNAs [45, 46]. These results suggest that additional variables, such as antibody designs (e.g. targeting different epitopes, full-length or scFv), cell types, and the processes of antibody internalization and endocytosis, can all contribute to the efficacy of AOCs.

### RNAs as therapeutic targets for antibody-oligonucleotide conjugates

#### Types of therapeutic oligonucleotides

The topics of therapeutic ONs have been extensively covered by other excellent reviews (siRNA [8]; ASO [47]; and others [11]). Here, we will briefly describe ON choices as payloads for AOC development (Fig. 1), including siRNAs, ASOs, aptamers, microRNAs (miRNAs), and CpG ONs. Because siRNAs and ASOs are more widely used in clinical and preclinical applications [11], the reported AOCs mainly utilized these two types of ONs.

siRNAs degrade complementary mRNAs through the RNA-induced silencing complex (RISC), mainly in the cytoplasm. Due to the bio-instability of siRNAs, they require heavy chemical modifications for clinical applications [48]. The phosphorothioate (PS) backbone is a common modification that enhances nuclease resistance in serum or circulation. Chemical modifications are also often introduced at the 2′-hydroxyl group of siRNAs, such as 2′-fluoro, 2′-*O*-methyl (2′-OMe), and 2′-methoxyethyl (2′-MOE), to enhance target RNA binding.

Similarly, ASOs often bear backbone modifications such as PS to increase stability, enhance cellular uptake, and promote target RNA engagement [47]. Chemical modifications at the 2′-hydroxyl group significantly improve ASO efficacy; prominent examples include 2′-OMe, 2′-MOE, locked nucleic acid, and constrained ethyl [47, 49]. ASOs act through two primary mechanisms. The first is RNase H-mediated degradation of target RNA. This type of ASO often has a gapmer structure: a full PS backbone or a mixed-backbone [50], with modified nucleotides (nt) at both ends flanking a central ~10 nt DNA core. Because RNase H exists in both the cytoplasm and the nucleus [51], ASO gapmers work in both cellular compartments, and are more effective than siRNAs in the nucleus. An ASO gapmer is exemplified by a gapmer ASO targeting the intron of the *C9orf72* RNA for treating amyotrophic lateral sclerosis [50]. The second mechanism is steric blockade, in which ASOs physically block ribosomal binding or splicing machinery to inhibit mRNA translation or splicing. These ASOs bear a full PS backbone and 2′-hydroxyl modifications throughout the entire sequence, unlike gapmers, and their binding with target RNA will not activate RNase H. A critical example is Spinraza, which disrupts pre-mRNA splicing of the *SMN2* gene for treating spinal muscular atrophy (SMA) [26].

Other ONs, such as miRNAs and aptamers, are also inhibitory modalities that can reduce target RNA levels. The FDA has approved two aptamers for the treatment of ocular diseases [52]. However, less progress has been made in using them as payloads for AOCs, partly because their drug targets are extracellular rather than intracellular. Conjugation of aptamers to antibodies could enhance their biodistribution, reduce the delivery barrier, and expand their therapeutic applications.

In contrast to target-inhibiting ONs, small activating RNAs (saRNAs, also known as RNAa) are a unique category of ONs that can activate gene expression [53]. These ONs have been reported to transcriptionally activate genes by assembling transcriptional activation complexes at target gene promoters [54, 55]. These ONs have shown promising clinical progress, e.g. MTL-CEBPA is the first saRNA in clinical trials for liver cancer by activating CCAAT/enhancer-binding protein alpha (C/EBP-α) expression [56]. While no reported AOCs have yet used saRNAs as payloads, this is a promising area for modulating disease processes by upregulating gene expression.

#### Targetable RNAs and RNA processing steps

Currently reported AOCs mainly target mRNAs for their degradation. For example, a polo like kinase 1 (*PLK1*)-siRNA/anti-HER2 AOC aimed to reduce *PLK1* mRNA to inhibit HER2+ breast cancer growth and metastasis [46]; AOC 1001 (del-desiran), the first AOC in a clinical trial to treat Myotonic Dystrophy Type 1 (DM1), acts via reducing the mRNA expression of a toxic form of the mutated myotonic dystrophy protein kinase (*DMPK*) gene in muscle cells [19].

AOCs could target an unlimited category of RNAs and RNA processing steps. First, given the success of ONs in splicing modulation [26], it would be convenient to use these ONs for AOCs as payloads. This is expected to ease the current ON delivery regimen, such as intrathecal injection to reach the CNS [26], which may also improve target knockdown efficacy [57]. Second, as compared to ADCs and small-molecule drugs, AOCs and their ON payloads are less sensitive to protein mutations and associated structural alterations (Table 1). This allows the development of AOCs to target gene mutants in diseases such as the *DMPK* gene mentioned above for DM1 [19], and potentially key drivers of cancer (e.g. KRAS or its mutants [25]) or neurodegeneration [e.g. microtubule-associated protein tau (*MAPT*) that encodes tau or its mutant [58, 59]]. Third, AOCs and their ONs have the unique advantage of targeting the mRNAs of genes that are traditionally “undruggable” for small molecules or ADCs, such as TFs [60]. For example, ASOs targeting *MYC* [24] can be a promising option for AOC development to target this common tumor-promoting TF. Fourth, in addition to protein-coding mRNAs, there are tremendous types and numbers of ncRNAs that are increasingly recognized to be important in diseases, and are suitable as drug targets [27, 61], such as long noncoding RNAs (lncRNAs), chromatin-associated RNAs (caRNAs), circular RNAs (circRNAs), tRNAs, small nucleolar RNAs, small nuclear RNAs, miRNAs, piwi-interacting RNAs (piRNAs), and many others. For example, metastasis-associated lung adenocarcinoma transcript 1 (*MALAT1*) is a lncRNA with known roles in cancer metastasis [62]. In fact, ASOs targeting *MALAT1* have shown potent effects in preclinical studies of metastasis [63, 64] and in modulating tumor microenvironment (TME) [65]. There are caRNAs generated from tissue-specific genetic elements such as enhancers (i.e. enhancer RNAs (eRNAs)), which display a high degree of tumor specificity [66, 67], and ASOs can effectively target them to inhibit oncogene expression or cell growth [67]. Similarly, circRNAs emerge as suitable druggable targets for a series of human diseases [68]. ASOs targeting these disease-associated RNAs, when coupled with disease-specific antigen/antibody pairs, can serve as ideal targets for AOC development.

### Conjugation strategies

Due to payload difference, AOCs differ significantly from conventional ADCs in conjugation strategies. ADCs typically attach hydrophobic small-molecule payloads to mAbs via covalent bonds [7, 69]. In contrast, ON payloads for AOCs bear large molecular sizes (~4–15 kDa, Fig. 1), high negative charges, and complex secondary structures, which influence the mAb’s isoelectric point [70], solubility [71, 72], and coupling orientations [73]. Consequently, among the key features for bioconjugation (i.e. electrostatic, steric, and hydrophobicity [74]), AOC chemistry generally prioritizes the first two parameters.

**Figure 2:**
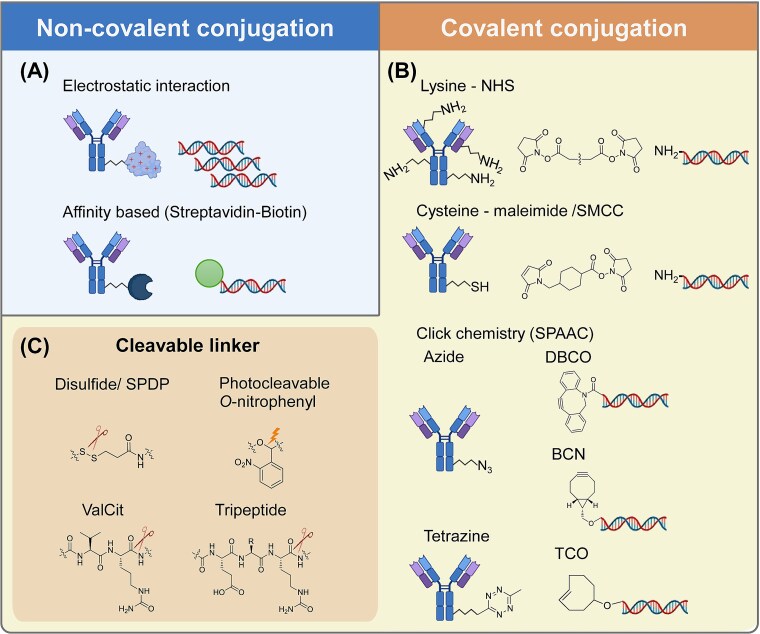
Common conjugation strategies for AOCs; noncovalent conjugation, in which ONs are associated with antibodies via electrostatic or affinity-based interactions, enables reversible complex formation; two illustrative examples are shown; covalent conjugation, involving stable chemical linkages between antibody and ON via reactive amino acid residues (e.g. lysine, cysteine) or site-specific modification; several common methods and conjugation chemistry are shown; BCN, bicyclononyne; DBCO, dibenzocyclooctyne; SMCC, *N*-succinimidyl 4-(*N*-maleimidomethyl)cyclohexane-1-carboxylate; TCO, trans-cyclooctene; NHS, *N*-hydroxysuccinimide ester; cleavable linkers can enable controlled release of the ON payloads from AOCs in response to specific intracellular stimuli such as enzymatic cleavage, redox potential, and photolysis; four examples of cleavable linkers and their cleavage site are shown.

The choice of conjugation methods impacts many crucial AOC characteristics, such as drug-to-antibody ratio (DAR), homogeneity, payload release, AOC stability, PK/PD, and therapeutic efficacy. We will discuss the two types of conjugation strategies below: noncovalent and covalent methods (Fig. 2).

#### Noncovalent conjugation

Noncovalent conjugation uses either electrostatic interactions or affinity-based methods, which link a mAb and ONs through reversible interactions, allowing facile preparation and tunable stability [75] (Fig. 2A). These constructs are prepared under mild conditions, preserving target-binding of both mAbs and ON payloads [76].

Electrostatic conjugates involve interactions between positively charged domains of an mAb with the negatively charged phosphate backbone of ONs [77]. Early AOC constructs used cationic sequences, such as protamine [45], poly-L-lysine [78], or cell-penetrating peptides [79]. Protamine has served as an effective bridge for ON conjugation [45, 46, 77, 80], e.g. cetuximab–protamine–siRNA achieved effective *KRAS* (G13D) gene knockdown (>80%) in colorectal cancer cells and detectable tumor inhibition in xenograft mouse models [77]. However, these constructs possess low *in vivo* stability and poor homogeneity, which limits manufacturing and clinical translation [18, 44].

Affinity-based noncovalent conjugation is exemplified by biotin–avidin, streptavidin–anti-avidin mAb, or engineered protein–ligand pairs [81], providing greater structural definition and homogeneity of AOCs. Such high-affinity interactions between mAb and ONs offer more consistent biophysical characteristics and functions compared to the electrostatic AOCs. For instance, a reported biotin-avidin mAb-siRNA conjugate achieved over 90% gene knockdown in HEK293 cells [82].

#### Covalent conjugation

Covalent conjugation creates stable bonds between mAbs and ONs, ensuring structural integrity, defined stoichiometry, and predictable performance *in vivo* (Fig. 2B) [34]. The chemistry has evolved from stochastic coupling (i.e. randomly occurring at reactive amino acid residues) to site-specific orthogonal conjugation.

##### Stochastic conjugation

Early AOC constructs adapt covalent conjugation methods used for ADCs, primarily amine- and thiol-reactive linkers. The stochastic reactivity of these chemistries causes structural heterogeneity and reduced antigen binding [83].

Lysine–*N*-hydroxysuccinimide ester (NHS) coupling modifies exposed lysine residues [83] with NHS-activated linkers bearing another reactive handle, such as maleimide [21], thiol [84], azide [85], tetrazine [86], or hydrazine [87] for subsequent coupling to ONs. Walker *et al*. used this approach to attach thiol-terminated ONs to anti-TfR1 mAb [21], improving activity but producing a variable DAR [75, 88], and reducing antigen-binding [89, 90]. Cysteine–maleimide coupling may offer better DAR control through interchain disulfide bond modification [91]. However, these AOCs were heterogeneous and susceptible to linker hydrolysis and retro-Michael addition reactions *in vivo* [91, 92].

##### Site-specific conjugation

Site-specific conjugation improves molecular homogeneity and therapeutic consistency of AOCs. Specific cysteine residues within human IgG1, such as V205C [92], can be engineered to allow thiol-maleimide coupling. Such AOCs maintained antigen binding, achieved 50%–70% mRNA knockdown *in vitro*, and successfully induced targeted gene silencing *in vivo* [44]. The β-lactam-lysine platform uses a reactive lysine at K99 in mAbs to enable conjugation with β-lactam-modified ONs [93], which generated an AOC with DAR 2 and effective target gene (*CTNNB1*) knockdown in cultured cells [93]. Additionally, enzymatic conjugation methods such as Sortase A and microbial transglutaminase (MTGase) enable site-specific bioconjugation under mild conditions without altering mAb structure or binding affinity. Sortase A links a terminal LPXTG tag and tetra- or penta-glycine-functionalized ONs [94], while MTGase couples glutamine residues (Q295) or a terminal Q-tag (LLQA or LLQGA) to amine-modified ONs to form stable amide bonds [95].

Most clinical-stage AOCs are constructed by site-specific conjugation to ensure homogeneity and stability (Table 2; see also Section *Clinical progress of antibody-oligonucleotide conjugate development*). Avidity Biosciences [34], Dyne Therapeutics [96], Tallac Therapeutics [97], and Denali Therapeutics [57] develop diverse conjugation platforms to generate homogeneous AOCs with DARs of 1 or 2. The success of these programs highlights the importance of the homogeneity to translate AOCs into clinical stages.

**Table 2:** AOCs under clinical trials show key information such as technology platforms, disease indications, clinical trial IDs, conjugation methods, and DAR.

AOC name/code	Company	Disease	ON target	mAb target	Trial ID/status (as of Dec 2025)	Description	Conjugation method	DAR
Del-desiran (AOC 1001/delpacibart etedesiran)	Avidity Biosciences	DM1	*DMPK* mRNA	TfR1	NCT06411288 (Phase 3, active) NCT05479981 (Phase 2, completed) NCT05027269 (Phase 1/2, completed)	TfR1-targeting mAb–siRNA (proprietary TfR1 mAb platform)	Site-specific cysteine-maleimide	~2
Del-brax (AOC 1020/delpacibart braxlosiran)	Avidity Biosciences	FSHD	*DUX4* mRNA	TfR1	NCT07038200 (Phase 3, recruiting) NCT05747924 (Phase 1/2, active)	TfR1-targeting mAb–siRNA (proprietary TfR1 mAb platform)	Site-specific cysteine-maleimide	~2
Del-zota (AOC 1044/delpacibart zotadirsen)	Avidity Biosciences	DMD	*DMD* mRNA (Induce Exon 44 skipping)	TfR1	NCT06244082 (Phase 2, active) NCT05670730 (Phase 1/2, complete)	TfR1-targeting mAb–PMO (proprietary TfR1 mAb platform)	Site-specific cysteine-maleimide	~2
Z-basivarsen (DYNE-101)	Dyne Therapeutics	DM1	*DMPK* mRNA	TfR1	NCT05481879 (Phase 1/2, recruiting)	TfR1-targeting Fab–siRNA (FORCE platform: TfR1 Fab fragment)	Site-specific thiol-maleimide	1
Z-rostudirsen (DYNE-251)	Dyne Therapeutics	DMD	*DMD* mRNA (Induce Exon 51 skipping)	TfR1	NCT05524883 (Phase 1/2, active)	TfR1-targeting Fab–ASO (FORCE platform: TfR1 Fab fragment)	Site-specific thiol-maleimide	1
TAC-001 (mozistobart zoratolimod)	Tallac Therapeutics	Advanced solid tumors	TLR9 receptor	CD22	NCT05399654 (Phase 1/2, active)	CD22-targeting mAb–CpG (Enzymatic/TRAAC platform)	Not cysteine-based	2
DNL628 (OTV:MAPT)	Denali Therapeutics	AD	*MAPT* mRNA	TfR1	NCT07328451 (Phase 1b, approved)	TfR1-targeting mAb–ASO Engineered TV to ASO via proprietary OTV platform	Chemical conjugation	~1

### Linker

#### Criteria for an “ideal” antibody-oligonucleotide conjugate linker

An ideal AOC linker should maintain circulation stability while enabling effective intracellular activity of the ON payload. In practice, this requires minimizing premature cleavage and exchange reactions in plasma, e.g. classical maleimide–thiol linkers can undergo retro-Michael deconjugation [98–100]. In addition, the linker must be compatible so that ON payloads can efficiently reach the required site of action (e.g. cytosol for siRNA and nucleus for ASOs and PMOs). Ideal linkers may also be designed to maximize endosomal escape, the dominant delivery bottleneck for ONs [16, 101]. Finally, linker choice should support developability and clinical translation, including controllable ON loading distributions, stability through purification, and high-fidelity bioanalysis of intact conjugates and relevant catabolites [102].

#### Noncleavable versus cleavable linkers

Noncleavable linkers are common for AOCs in early attempts because they are chemically robust, easy to develop, and less prone to premature payload release [18, 73]. Such AOCs have displayed *in vivo* activity in many settings [103]. The main risks of noncleavable linkers are: (i) steric hindrance (derived from the intact mAb), and (ii) persistent linker remnants on the ONs (after mAb degradation), which can reduce their functional RNA engagement. These risks increase with higher DAR and via stochastic conjugation. In contrast, cleavable linkers can be designed to release ONs from AOCs only in intracellular spaces (Fig. 2C). However, they also pose a key trade-off similar to ADCs: overly increased linker stability can reduce intracellular cleavage [104, 105]. In AOCs, cleavable designs commonly use sterically tuned disulfides or protease-cleavable peptides (e.g. Val-Cit) (Fig. 2C). Unlike ADCs, cleavable linkers are not yet a default AOC strategy, and available data suggest that their benefits are context dependent. In a structure–activity relationship (SAR) study of an antibody–siRNA conjugate, a Val-Cit cleavable linker did not show a clear advantage over stable noncleavable linkers, consistent with the possibility that intracellular processing of the latter may yield siRNA-derived catabolites that contain linker remnants yet still work well for RISC loading and RNA knockdown [71]. Beyond canonical triggers, a photocleavable linker has been employed to enable spatially controlled siRNA release. There are also modular cathepsin B-cleavable linkers that can be utilized for AOCs where traceless payload liberation is advantageous [106].

#### Linker length, polarity, and charge management

In ADCs, linker design mainly considers payload hydrophobicity and propensity to aggregation [107]. In AOCs, by contrast, electrostatics and charge density of linkers are more important constraints. As such, higher DAR and charge clustering of ONs can accelerate AOC clearance. Steric or electrostatic effects can also impair target engagement, consistent with observations that excessive drug loading can reduce functional antibody binding [103].

The length of the linker (or spacer) is therefore crucial to reduce ON charge clustering and steric crowding, improve solubility, and limit nonspecific interactions. Polyethylene glycol-containing spacers are commonly adopted in this context, given the established impacts of PEGylation in ON delivery [108]. However, linker length and polarity require balance: very short linkers can exacerbate clustering and steric interference, whereas overly long or bulky spacers may reduce stability or perturb receptor binding and internalization [71, 73].

#### Linker choice depends on modality and intracellular mechanism

Linker requirements differ across AOCs because each ON category engages distinct intracellular machinery with different tolerance to steric bulk, residual linker remnants, and subcellular misrouting [18]. Constraints are often most stringent for siRNA AOCs, which require cytosolic delivery and RISC loading, consistent with *in vivo* SAR showing dependence on conjugation site, loading, and siRNA chemistry [71]. For ASO gapmers, linker optimization is typically driven by nuclear delivery and steric accessibility for target hybridization and RNase H1-mediated cleavage [11]. Splicing-switching PMOs often tolerate stable linkers and residual fragments [103]. Together, these results indicate that linker architecture, ON conjugation site, ON subtypes and features, and DAR should be tailored to the required intracellular site of action and to the steric tolerance of the relevant RNA processing machinery.

Available studies indicate that conjugation strategy and linker selection critically influence AOC PK and efficacy in a manner dependent on ON payloads (see a summary in Table 3). However, systematic and head-to-head comparisons of these design parameters remain limited, representing a significant knowledge gap in the field. Addressing this gap through well-controlled studies across diverse mAbs and ON payloads will be essential for enabling rational AOC design and optimizing therapeutic performance.

**Table 3:** AOC designs in consideration of classes and properties of ON payloads, i.e. their subcellular destination and mechanisms of action dictate linker choice, PK risk, and optimal DAR.

Payload class	Subcellular destination	Linker requirements	Major PK risks	References
siRNA	Cytoplasm	VCit cleavable linkers show similar PK to noncleavable linkers Conjugation at either the 3′ or 5′ end of the sense strand does not significantly impact PK and target knockdown Residual linker does not impair RISC loading	Higher DAR is associated with reduced plasma exposure Disulfide linkers may cause premature payload release and reduced PK	[71]
ASO (knock down)	Nucleus and cytoplasm	Limited systematic evaluation of linker requirements	PK behavior remains incompletely understood Higher DAR may increase nonspecific hepatic uptake	[109]
PMO	Nucleus	No significant difference in PK or exon skipping between cleavable and noncleavable linker Conjugation at either the 3′ or 5′ end of the PMO does not affect exon skipping	DAR appears to have minimal impact on PK	[103]
CpG	Endosome	Linkers must maintain stability in circulation to avoid premature payload release	PK and biodistribution remain insufficiently characterized	[110, 111]

## Antibody-oligonucleotide conjugate fates in circulation, during and after cellular entry

**Figure 3:**
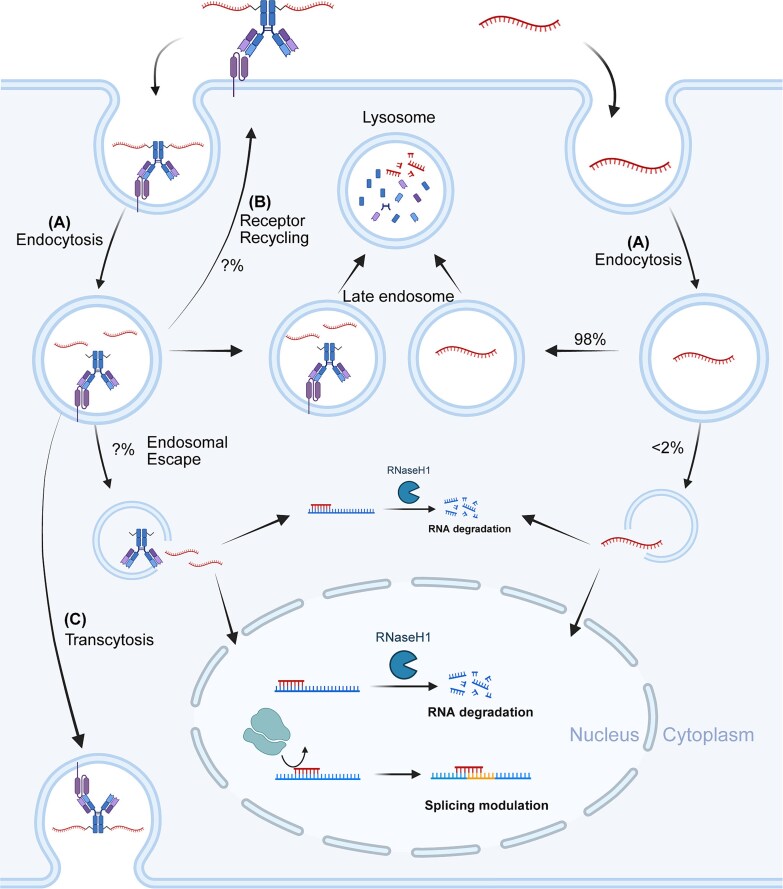
Comparison of the fates and action mechanisms of ASO-loaded AOCs after cellular entry (left half), as compared to unconjugated ASOs (right half). (A) Endocytosis: AOCs enter cells primarily through receptor-mediated endocytosis (the left half), whereas unconjugated ASOs (the right half) are internalized mainly via nonspecific uptake pathways. (B) Receptor recycling: following internalization, AOCs may undergo receptor recycling, during which the antibody–receptor complex is trafficked back to the cell surface and released. (C) Transcytosis: certain AOCs can exploit receptor-mediated transcytosis pathways to cross biological barriers such as the BBB, facilitating tissue access not achievable by unconjugated ASOs; question marks in the left half denote that it is not well known how AOCs undergo receptor recycling, and limited information is available about the percentage of their escape from the endosome (created in BioRender).

The mechanism of action of AOCs is often presumed to be similar or identical to that of ADCs or unconjugated ONs (intracellularly) (Fig. 3). However, limited research results are available to make concrete conclusions. We summarize the general knowledge from these limited results, making some assumptions and speculations. Dedicated work is warranted to fully understand AOCs’ PD, stability in circulation, cellular uptake processes, dynamics, and fates after internalization, and how these impact target perturbation or therapeutic efficacy (Fig. 3).

### Antibody-oligonucleotide conjugates in circulation and current knowledge of their pharmacokinetics

Circulation half-life follows a general trend: mAb (weeks) > AOC (4–12 hr) ≫ ON (<0.5 hr), illustrating that antibody conjugation substantially extends the plasma stability of ONs [57, 103, 112, 113]. Free ONs are typically cleared through renal filtration. In contrast, conjugation to mAb alters their biodistribution and shifts clearance from renal to hepatic pathways [22, 71, 103]. A direct comparison among mAbs, AOCs, and free ONs is rare, but available studies support this general trend. For instance, oligonucleotide transport vehicle (OTV), an OTV system, engineered to express an antibody that binds to human TfR1 via the Fc domain, exhibited markedly prolonged plasma half-life compared to unconjugated ASO in mice [57]. Conversely, increasing ON loads on the antibody can negatively impact antibody PK: an anti-TfR1-PMO AOC with a DAR of 2 showed accelerated clearance and lower plasma concentrations than the naked antibody [114].

The PK of AOCs can be critically affected by DAR, ON payloads, conjugation strategies, molecular sizes and modifications of mAbs, and linker chemistry. Higher DAR could theoretically deliver more ON payloads, potentially improving AOC efficacy. But unexpectedly, a recent study showed that a siRNA-AOC with DAR 3 was cleared from plasma more rapidly than that with DAR 1 [71]. This was attributed to increased negative charge and a higher PS content of more ON payloads, which promote hepatic uptake while reducing delivery to the intended target tissue. However, for AOC with neutral-charge payloads such as PMO, an increase in DAR to 4 did not appear to affect plasma half-life [103]. Conjugation strategy also modulates AOC PK. In one study, lysine- and glycan-based conjugation of siRNA-AOCs resulted in faster plasma clearance as compared to cysteine-based conjugation, whereas random cysteine conjugation showed comparable clearance to site-specific cysteine approaches [71]. Together, these findings highlight that both the chemical properties of the ONs and the conjugation strategies critically shape AOC PK.

The size of the mAb impacts the PK of AOCs. Smaller fragments, including Fab (~50 kDa), scFv (~25–30 kDa), and nanobodies (~15 kDa), offer improved tissue permeability owing to their reduced size and quicker diffusion kinetics [115]. Yet these smaller constructs generally lack the Fc region and are not recycled via neonatal Fc receptor (FcRn), so they tend to be cleared more rapidly [116].

In addition, modifications of the mAb also affect AOCs’ PK. For example, PEGylation of AOCs can increase serum stability and enhance tissue distribution and efficacy [71], which works by shielding the conjugate from enzyme degradation, reducing nonspecific protein binding, and decreasing renal clearance [108, 117]. Recently, an alternative shielding strategy has been reported in which sequence-independent ASO-binding antibodies were incorporated into AOC designs to cloak the polyanionic ASO payload, resulting in improved plasma PK, reduced nonspecific cellular interactions and higher target tissue delivery [113].

Future studies should perform robust, side-by-side comparisons of the PK, clearance, and stability of AOCs, ONs, and mAbs in matched platforms, while systematically evaluating how antibody format, linker chemistry, DAR, and ON type influence AOC behavior in circulation and therapeutic efficacy.

### Cellular uptake, receptor-mediated internalization, and endosomal escape

There are three main routes of AOCs intracellularly, as shown in Fig. 3, which are: (A) internalization into the endosome-lysosome pathway in the targeted cells; (B) recycling out of the cells; and (C) transcytosis out of the cells into hard-to-penetrate tissues. Route C is required explicitly for crossing biological membranes (e.g. TfR1-mediated CNS delivery across the BBB) (Fig. 3C). Once an AOC has completed BBB crossing, it will still need to undergo route A to enter the target cells. In general, improving route A can enhance ON drug delivery and therapeutic efficacy in target cells, whereas route B can negatively affect AOC effects.

Our knowledge of antigen binding and intracellular fates of AOCs remains limited. Reported work indicated that the features of ON payloads can affect their cellular entry and intracellular fates. One study showed that at DAR 1, ASO conjugation did not impact antibody binding to the antigen (TfR1) or its internalization [57]. In contrast, in another study, AOCs with higher DAR values could impair antigen recognition [103]. Intriguingly, in the same study the ultimate biological activity of the AOC—the exon-skipping efficacy—was still enhanced [103], indicating that AOC efficacy is determined by a tug-of-war between DAR, antigen-binding affinity, and internalization, among other factors. Notably, some antibody–antigen pairs that work well for ADCs may not work well for AOCs, e.g. in the Cuellar *et al*. [44] study, the authors found that siRNA-anti-HER2, one of the best-performing antibodies for ADCs, did not achieve effective target RNA knockdown. In addition to impacting antigen binding, the “bulky” size and charge of ONs could also impact receptor recycling (Fig. 3, route B), which, to our knowledge, has not been tested. For unconjugated ONs, once internalized into cells, most are retained in endosomes, where they cannot be released into the cytoplasm, and thus cannot achieve target perturbation [101] (Fig. 3, right side). AOCs, according to limited reports, still appear to suffer from this critical endosome entrapment. For example, Barker *et al*. compared anti-TfR1 AOC with unconjugated ASO payloads, finding no difference in ASO concentrations in early/late endosomes, lysosomes, or in the nucleus 24 hr post-treatment, although AOCs may reach concentrations faster than unconjugated ASO [57]. In summary, AOC efficacy depends not only on antigen binding but also on intracellular trafficking and endosomal escape, which remain major bottlenecks.

One important concept about AOCs is that they are compositionally complex biologics contrasting traditional single-component agents. This is because the behavior of AOCs is strongly influenced by the antigens/antibodies, ON payloads, and conjugation strategy, and each of these three components can be highly variable. In practice, AOC performance is governed by multiple interdependent factors, including antigen accessibility, intracellular trafficking, site of action, payload-specific linker design, and DAR. Unfortunately, comprehensive studies of AOC features based on ON classes and linker chemistry remain limited. We summarize the currently available results about optimal linker strategies and key AOC design considerations, as well as associated risks derived from each ON payload class to help illustrate some key distinctions (Table 3).

## Therapeutic applications

AOCs have shown promising effects across several indications, which include neurological diseases, muscle disorders, rare genetic diseases (RGDs), and oncology. There are also studies related to infectious diseases, such as HIV [45, 118], but we will not dedicate a separate section to them.

### Neurological diseases

ON drugs, particularly ASOs, have already shown strong promise for treating neurological diseases, as exemplified by Spinraza. However, intrathecal injection is common but has shortcomings: it is invasive, diffusion-mediated, and inefficient in reaching deep brain regions [119–121]. AOCs hold advantages for neurological diseases because: (i) conjugation of ONs to BBB-penetrating antibodies, such as anti-TfR1, can facilitate ONs crossing the BBB (route C in Fig. 3); (ii) upon entering CNS, antibodies may also promote cellular uptake of ONs into specific cell types that highly express the target antigens (route A/B in Fig. 3); and (iii) the treatment regimen for AOCs can be less invasive, e.g. by intravenous infusion (I.V.). Notably, antibodies that promote BBB penetration are not necessarily effective for facilitating cellular uptake. For example, the reported anti-TfR1 antibody used in the OTV platform is effective in the former task, but does not obviously increase ASO uptake in neurons relative to ASO-free uptake [57].

AOC development for neurological diseases is exemplified by a study that conjugated a murine anti-TfR1 antibody to a PMO to modulate splicing of *SMN2* [114]. In this work, the systematic delivery of DAR 2 AOC elevated the full-length *SMN2* mRNA levels in the CNS, leading to extended survival in a severe SMA mouse model. Another example is the I.V. delivery of OTV with an ASO targeting *MALAT1*, which achieved uniform ASO biodistribution in the mouse CNS, resulting in more significant *MALAT1* RNA knockdown in the CNS than an AOC using a non-TfR1-binding antibody or ASO-only [32].

### Muscular diseases and rare genetic disorders

Muscular diseases are common indications for in-development AOCs. This is due to the availability of both suitable antibodies and antigens, as well as known causal genes for such diseases. One example is an siRNA-AOC targeting *myostatin* (*Mstn*) using an anti-CD71 Fab that achieved durable gene silencing in cardiac and skeletal muscle in mice [91]. Similarly, AOCs conjugating anti-TfR1 mAbs to various ONs (siRNA, ASOs, and PMOs) demonstrated efficient delivery to muscle tissue in both mice and nonhuman primates [34]. Notably, the potency of knockdown is most pronounced in muscle compared to other tissues, demonstrating tissue specificity of these AOCs. These findings lay the groundwork for the first AOC drug in clinical trials for the treatment of DM1.

AOCs hold promise to address many other RGDs beyond muscle disorders. Because of their rarity, RGDs are not popular targets for small-molecule drugs due to the high cost and long duration of development [122, 123]. In contrast, precision or personalized AOC medicine could be a perfect fit for RGDs, given its relatively straightforward target selection and ON design, as well as likely shorter development times and lower costs.

### Oncology

In oncology, AOCs offer a novel strategy to manipulate cancer or cancer-neighboring immune cells by targeting tumor-specific antigens and druggable RNA targets. A study in 2005 pioneered the potential of AOCs for cancer therapy [45]. The authors fused antibodies to protamine, enabling conjugation to siRNAs at an estimated DAR of six. This AOC prototype successfully delivered siRNAs to tumor sites and inhibited the growth of the B16 melanoma model [45]. The protamine strategy was subsequently also used by other studies to conjugate anti-epidermal growth factor receptor (EGFR) mAb [77] or anti-HER2 mAb [46] with siRNAs targeting cancer driver genes (mutant KRAS and PLK1), which showed *in vivo* efficacy.

In other cancer types, such as glioblastoma, antibodies targeting CD44 and EphA2 deliver an ASO to reduce FAM107A expression, a driver of tumor invasion, providing a promising strategy for treating this highly aggressive brain tumor [124]. For precursor B-cell (pre-B) acute lymphoblastic leukemia, MYC-associated factor X dimerization protein 3 (MXD3) is required for cancer survival [125]. Satake *et al*. developed an anti-CD22 mAb/*MXD3*-ASO conjugate, achieving significant tumor inhibition and extended mouse survival [126]. In pre-B leukemia driven by the *E2A–PBX1* fusion gene, AOCs can employ anti-CD19 mAb to deliver ASOs that specifically target this driver gene, demonstrating the dual selectivity conferred by AOCs [127].

AOCs have both advantages and disadvantages as compared to ADCs in oncology (Table 1). ADC payloads are often highly cytotoxic, and thus any off-tissue binding of the antibody could be detrimental (e.g. an anti-EGFR mAb may cause dermatologic toxicity [128]). In this regard, ON payloads can be designed to target cancer-overexpressed, cancer-mutated, or cancer-specific driver genes that are absent or nonessential in normal cells, thereby avoiding off-tissue toxicity. On the flip side, AOCs’ lower toxicity may also be disadvantageous for oncology, as ONs act by modulating specific genes rather than killing cells. This means that their inhibitory effect on tumor growth may be chronic and moderate as compared to that of cytotoxic payloads in ADCs.

Beyond direct modulation of cancer cells, AOCs can also be used to reprogram the TME by delivering immunostimulatory ONs. For example, CpG-containing ONs can activate toll-like receptor 9 (TLR9) in B cells and myeloid cells, thereby enhancing innate and adaptive antitumor immune responses. Conjugating CpG ONs to immune cell-targeting antibodies enables localized immune activation within the TME, reducing the need for intratumoral administration required for free CpG ONs [110, 111, 129].

### Clinical progress of antibody-oligonucleotide conjugate development

Several leading companies have made notable clinical progress on AOCs. A detailed list of assets has been provided by other reviews [127, 130, 131]. We only summarize the key highlights here while noting that many findings remain preliminary or only from the early phase of trials.

Avidity is developing AOCs for treating several genetic diseases, with a focus on muscle disorders. They have AOCs in clinical trials for indications such as DM1, facioscapulohumeral muscular dystrophy (FSHD), and Duchenne Muscular Dystrophy (DMD) (Table 2). Among others, Delpacibart etedesiran (del-desiran) is designed to target the toxic *DMPK* mRNA driving DM1 pathology, which consists of a siRNA conjugated to anti-TfR1 mAb. In the Phase 1/2 MARINA study, treatment led to ~ 40% mean reduction in *DMPK* mRNA across participants (*n* = 38), with an acceptable safety and tolerability profile [132]. In the Phase 2 open-label extension (MARINA-OLE), the AOC continued to show favorable safety and preliminary signals of clinical benefit in 37 patients. While these findings are encouraging, they are derived from relatively small cohorts and remain under ongoing evaluation. Based on these results, del-desiran has advanced into a Phase 3 trial (HARBOR), with topline efficacy data anticipated in Q2 2026.

Similarly, Dyne Therapeutics is working on AOCs for DMD and DM1 (Table 2). Their platform utilizes an anti-TfR1 Fab to deliver a *DMPK*-targeting ASO for DM1 and an exon51-skipping PMO targeting the same gene for DMD. Instead of full-length antibodies, they use a Fab format and intended to enhance tissue penetration, improve tolerability, and reduce the risk of immunogenicity. Notably, positive topline data from the Phase 1/2 study of z-rostudirsen demonstrated a favorable, sustained safety and tolerability profile in a cohort of 86 patients, along with an approximately seven-fold increase in muscle content at 6 months, accompanied by early functional benefits to the patients. However, these outcomes are from the early phase of the trial and require further validation in larger and longer-term studies.

Tallac Therapeutics focuses on Toll-like Receptor Agonist Antibody Conjugates (TRAACs). Their research leverages an anti-CD22 mAb to deliver CpG ONs that activate the TLR9 receptor (TAC-001, Table 2). This approach is designed to stimulate the B-cell immune responses in the TME, thereby enhancing the efficacy of cancer immunotherapies. TAC-001 is currently in a Phase 1/2 clinical trial. Preliminary data indicate that it is well-tolerated and shows early clinical activity, supporting the feasibility of AOCs as a strategy to enhance cancer immunotherapy.

These clinical examples highlight both the progress achieved and the substantial potential of AOCs across diverse disease indications. It is noteworthy that the development of AOCs has primarily focused on muscle disorders and well-characterized genetic diseases with known causal genes. The emphasis on muscle indications is partially driven by the availability of well-established targets such as TfR1 and corresponding antibodies suitable for AOC design. Expanding the identification of appropriate antigens, antibodies, and druggable genes or RNAs in other tissues will be critical to advancing the clinical development of AOCs.

## Challenges and limitations

AOCs face several key challenges that need to be addressed to achieve widespread clinical success. Among others, these include endosomal escape, immune responses, off-target problems, and manufacturing issues.

### Biological understanding of antibody-oligonucleotide conjugate action is incomplete

#### Endosomal entrapment and intracellular delivery

A major challenge for ON-based therapeutics, including AOCs, is inefficient intracellular delivery [101]. Once AOCs bind target cells, they must undergo receptor-mediated endocytosis and then escape from the endosomes to release the ON payloads inside the cell. For unconjugated ONs, <1% can escape from endosomes to achieve therapeutic effects [101]. While not yet tested for AOCs, this percentage is estimated to be similar [57]. There are strategies to enhance ONs’ escape, including the incorporation of endosome-disrupting agents, such as cationic polymers or fusogenic lipids, which interact with endosomal membranes to destabilize and facilitate payload release. Cationic polymers like polyethyleneimine [133] and fusogenic lipids such as dioleoylphosphatidylethanolamine [134] have demonstrated the ability to facilitate nucleic acid escape from endosomes. Additionally, modulation of endogenous lipid metabolism has been shown to affect endosomal membrane dynamics. Molecules such as lysobisphosphatidic acid [135, 136], free fatty acids, ceramide [137], and cholesterol [137] can alter membrane curvature and permeability, potentially promoting ON escape. These molecules can be incorporated into AOCs to facilitate the escape of ON payloads.

Small molecules have also been reported to promote ON escape. Compounds such as UNC10217938A [138] and SH-bc-893 [139] disrupt endosome trafficking, prevent lysosomal degradation, and prolong cytoplasmic ON persistence. However, the targets of these molecules and their mechanisms of action are not yet understood. Genetic modulation also holds promise for enhancing endosomal escape. Studies have identified specific genes, such as *Rab5C* [140] and *GOLGA8* [141], that are essential for endosomal trafficking and may promote the endosomal escape of ONs. These genes could be targeted to enhance AOCs’ efficiency.

#### Immune responses and off-target effects

AOCs could be recognized as foreign agents, leading to the development of ADAs that may neutralize the therapeutic effect or cause allergic reactions [142]. While typical heavy chemical modifications to ON payloads, such as siRNA and ASOs, may reduce innate immune sensing [143], the holistic AOC structure remains a potential target for immune surveillance.

AOCs can induce off-target effects if the antibody recognizes antigens expressed on unintended cells. In some cases, increasing the DAR may exacerbate nonspecific hepatic uptake, as the charge and size of ON payloads may alter the antibody surface properties and interfere with antigen recognition [103]. In one study, AOCs conjugated with single-stranded ONs showed more nonspecific interactions with cell-surface proteins than AOCs carrying double-stranded ONs, an effect likely due to the higher nonspecific binding propensity of single-stranded ONs [109]. Furthermore, the off-target toxicity of AOCs can also be caused by ON binding to unintended RNAs and proteins [49, 144–147].

### Chemistry and manufacturing challenges

#### Conjugation efficiency and scalability

A central challenge in AOC development is achieving efficient and reproducible conjugation of ONs to mAbs while maintaining defined payload stoichiometry, antibody functionality, and ON integrity. Site-specific conjugation strategies appear to reduce heterogeneity and improve batch consistency for ADCs [148, 149] and are now increasingly applied to AOCs to control the DAR and minimize variability [44]. This is exemplified by the THIOMAB cysteine engineering platform. For clinical translation, conjugation processes must be both reproducible and scalable. Conventional approaches relying on stochastic lysine modification or uncontrolled thiol coupling often generate heterogeneous products that complicate purification, downstream analytics, and regulatory compliance under cGMP standards [150, 151]. Accordingly, successful advancement of AOCs requires integrated optimization of conjugation chemistry, analytical methods, and process engineering. The implementation of site-specific conjugation platforms, robust DAR characterization assays, and cGMP-compliant process design will be essential for producing homogeneous and well-characterized AOCs at scale.

#### Steric effect of the ON payloads and linker

Increasing DAR has been widely pursued in ADCs to enhance potency. However, excessive loading can impair antigen binding [152]. A similar challenge exists in AOCs, where bulky and negatively charged ONs introduce steric hindrance that may reduce antigen engagement and cellular uptake. For example, PMO-based AOCs exhibit up to five-fold reduction in binding affinity at higher DARs, likely due to steric masking by the ONs [103].

Linker design also strongly influences AOC performance. Linkers must remain stable in circulation while enabling efficient intracellular release, often necessitating longer and more complex structures that increase molecular weight and steric burden. Residual linker fragments may impair ON hybridization or protein interaction (e.g. RISC) as observed in siRNA-conjugated AOCs [71, 103]. Traceless linkers, such as para-aminobenzyl group have been developed to ensure efficient and intact payload release [71, 106]. Overall, optimizing AOCs requires balancing DAR and linker design to maximize therapeutic efficacy while minimizing steric interference.

#### Novel physicochemical properties and hydrophobicity

Unlike ADCs, which often involve hydrophobic payloads driving aggregation and promoting *in vivo* clearance [107], AOCs incorporate large, highly negatively charged ONs that significantly alter antibody surface charge and colloidal behaviors. These differences introduce a distinct formulation challenge. Banks and Cordia conducted a detailed biophysical characterization of AOCs, showing that formulation parameters, such as pH and ionic strength, strongly affect colloidal stability, and that conditions optimized for conventional mAbs or ADCs are not directly transferable to AOCs [70]. Electrostatic interactions between negatively charged ONs and positively charged mAbs residues can promote aggregation and reduce stability. Adjusting ionic strength or pH can mitigate these effects [70]. These findings highlight the need for formulation strategies specifically tailored to the unique electrostatic and structural properties of AOCs.

#### Bioanalytical limitations

Comprehensive post-synthesis characterization of AOCs is essential but technically challenging due to their hybrid nature. Standard protein liquid chromatography (LC) methods are often incompatible with ONs, complicating purification and analysis. Although mass spectrometry (MS) can confirm molecular weight and conjugation of AOCs, it has limited accuracy for DAR determination due to variable ionization efficiencies across species [153]. This challenge is exacerbated in heterogeneous, stochastic conjugated products. Advanced analytical approaches, including size-exclusion chromatography–MS, strong cation-exchange chromatography–MS [154], anion-exchange chromatography–MS, size-exclusion chromatography-multi-angle light scattering [155], and specialized AOC LC–MS workflows [156, 157], are required to assess DAR distribution, conjugation homogeneity, and product purity. Establishing standardized analytical and purification protocols will be critical for ensuring reproducibility and regulatory compliance in cGMP manufacturing.

Beyond physicochemical characterization, evaluating the *in vivo* behavior of AOCs remains a major challenge. Current PK studies often quantify free ONs rather than intact AOCs, limiting insight into stability and biodistribution *in vivo* [34, 57, 114]. This reflects the difficulty of distinguishing the intact AOCs from the released payloads in the biological matrices. To address this gap, integrative analytical strategies need to be designed to simultaneously track free mAbs, free ONs, intact AOCs, and catabolites, which can be achieved by techniques such as hybrid enzyme-linked immunosorbent assay, advanced LC–MS workflow, and immunostaining. For example, immunostaining can examine both the antibody and the ASO payloads to monitor cellular and tissue distribution, including within the brain [57].

## Opportunities and future directions

Despite the challenges, AOCs remain an exciting therapeutic class with the potential to transform the treatment of virtually any human disease. We discuss below the opportunities that exist to improve the three major components of AOCs.

### Explore new antigens/antibodies and their engineering

**Figure 4:**
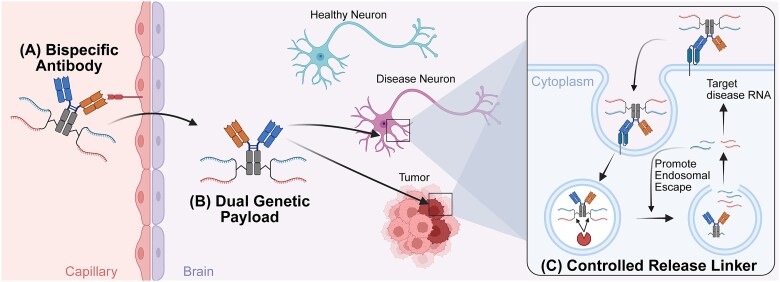
Future opportunities in AOC Research and Development; this diagram presents one hypothetical example of potential opportunities for future innovation across the three areas of AOCs: antibody engineering, linker chemistry, and target RNA selection. (A) Antibody engineering and multispecific formats, such as bispecific antibodies, can enable engagement of multiple antigens or receptors, thereby improving tissue targeting, cellular uptake, or transcytosis during delivery. (B) Dual genetic payload strategies, in which two genetic cargos are co-delivered to enhance therapeutic efficacy, enable synergistic gene modulation, or broaden the therapeutic window and disease scope. (C) Advanced conjugation strategies and linker engineering, including cleavable and traceless linkers, to achieve controlled intracellular release of ON payloads while minimizing premature release and off-target effects. The example with two colors of ASOs (blue and pink) aims to show a possible combination of payloads to target a disease–associated RNA and an endosomal regulator gene simultaneously (Created in BioRender).

The availability of validated antigens/antibodies is a constraint on the development of AOC drugs. Progress in cell surface epitope proteomics [158], disease transcriptomes [159], or the combination of the two [160] has offered an opportunity to explore new cell-type- or disease-specific-cell surface proteins that can serve as effective antigens for antibody development (Fig. 4A). Cancers are unique in harboring large numbers of neoantigens, many of which can be exploited as disease-specific targets for antibody and AOC development [161]. The rapid growth of artificial intelligence (AI) for epitope prediction and antibody design [162, 163] offers powerful new tools to catalyze this field.

In terms of antibody engineering, one opportunity for AOC development is to use bispecific or multispecific antibodies (Fig. 4A). For example, one can engineer a bispecific antibody with one Fab domain designed to facilitate transcytosis through the BBB (e.g. anti-TfR1). At the same time, the other Fab can be engineered to promote uptake by a specific CNS cell type (e.g. anti-triggering receptor expressed on myeloid cells 2 for microglia) [164, 165]. As our understanding of the mechanisms underlying AOCs’ intracellular fates increases, opportunities will arise to engineer antibodies to prevent rapid recycling and to selectively modulate their fates along the three routes shown in Fig. 3 to achieve the desired route.

### Other disease indications and antibody-oligonucleotide conjugate targets

Besides those discussed above, several other diseases are suitable for AOC development, which include neurodegeneration, liver diseases, fibrosis, and autoimmune disorders. A few ONs are already in clinical trials for neurodegenerative diseases such as Alzheimer’s disease (AD) [58]. It offers opportunities to conjugate such ONs to brain-penetrating mAbs, such as anti-TfR1 mAb, to facilitate CNS delivery (Fig. 4) [57]. It will be an excellent opportunity to develop further bispecific antibodies that not only help the AOC cross the BBB but also enhance its uptake by specific diseased cell types or even subtypes, such as neuron subtypes more vulnerable to AD [166, 167], or microglia that play key roles in AD [164, 165]. In the liver, while ONs can effectively reach hepatocytes, other liver cell types are not well targetable. For example, hepatic stellate cells are crucial for liver function and diseases such as liver fibrosis or cirrhosis, which impact a large population of patients [168, 169]. Promising antigens and antibodies have been identified for this cell type, potentially enabling the delivery of AOC drugs to treat fibrosis [170, 171]. Autoimmune disorders are also suitable for AOC development. For example, several ADCs have been reported to treat arthritis, which often conjugate antibodies that recognize overactive immune cells with payloads that can either kill these immune cells or functionally modulate immunity [172, 173]. These ADCs can inspire AOC development because effective antigen/antibody pairs are readily available, and the payloads in ADCs can be replaced with ON payloads. In this case, ON payloads offer advantages over small-molecule payloads due to their precision in targeting specific genes/mRNAs and the straightforward development process. In fact, one AOC has already been reported for treating arthritis in mouse models by conjugating anti-C5aR1 mAb with a C5-siRNA payload to knock down the complement component C5 [174].

The improved precision and efficacy of AOCs compared to traditional ON drugs will enable the exploitation of many more RNAs as drug targets. Current ON drugs mainly target mRNAs (wild type or mutant). There is a large category of noncoding RNAs with increasingly known functions (see Section *Design and engineering of antibody-oligonucleotide conjugate*). Among these, lncRNAs are promising targets due to their diverse roles in human diseases [61, 175]. We use two critical tissues, the brain and heart, as examples to illustrate the opportunities for AOCs. In the brain, lncRNAs can aberrantly regulate key gene expression involved in disease pathology, and their inhibition can alleviate disease phenotypes, as seen with *BACE1-AS* lncRNA [176] for AD and *UBE3A-AS* lncRNA [176, 177] for Angelman syndrome, respectively. In the heart, several lncRNAs have already been targeted by ASOs to benefit cardiovascular diseases, such as reducing atherosclerotic plaque and fibrosis [178, 179]. Going beyond knockdown, AOCs can deliver gene-activating ONs, such as saRNAs [53, 180]. Since many diseases result from the loss-of-function of a gene [181] or haploinsufficiency [182], AOC drugs that can restore gene expression in a tissue-specific manner will be an exciting opportunity with broad applications.

### Innovations in conjugation strategies and linker chemistry

A key opportunity for AOCs is to make structure control a routine rather than an exception. Relative to stochastic lysine coupling, site-specific conjugation provides tighter control over conjugation site and loading, simplifying manufacturing and reducing the risk that ON charge and size compromise antibody developability [18, 73], with more interpretable SAR.

Linker and spacer design for AOCs are lever for developability and delivery. Because ONs can introduce strong electrostatic interactions, spacers can manage charge clustering, minimize nonspecific interactions, and preserve formulation robustness, often in conjunction with systematic SAR workflows [70]. These efforts enhance mechanism-relevant analytics that distinguish intact AOCs, processed intermediates, and released (or still tethered) ON catabolites rather than relying solely on measuring total antibody exposure [102].

Cleavable linkers remain a high-upside but unsettled design space, primarily because the value proposition depends on achieving functional release of ONs without sacrificing systemic stability. Current *in vivo* comparisons indicate that canonical ADC-style cleavage does not confer a consistent advantage for AOCs [71]. Future compelling cleavable-linker opportunities will likely come from designs that align efficient cleavage with endosomal escape, so that bond cleavage becomes part of a coordinated route to productive cytosolic or nuclear delivery rather than an isolated chemical event [106, 183] (Fig. 4C).

### Multispecific targeting to simultaneously manipulate genetics and chemical biology

Multispecific drugs (MSDs) can specifically impact multiple drug targets, which are proposed as a significant innovation in biopharmaceutics [184, 185]. Within this context, AOCs belong to MSDs, or more precisely, dual-specific drugs (antibodies that bind specific antigens; ONs that recognize specific RNAs). Future endeavors can engineer AOCs to bear additional specificity. For example, it is possible to target two RNAs using two distinct ON payloads conjugated to a single mAb. This can be a combination of one splicing-modulating ASO with a second one that knocks down a mutated mRNA, or it could involve two ONs that simultaneously knock down two genes in the same cell (Fig. 4B). This latter case could be promising for targeting cancer, where synthetic lethality is common [186]. Moreover, built on a better understanding of genetic factors involved in endosome release, the AOC payloads can include one ON that manipulates the endosome release to benefit the other ON payload targeting a disease RNA (Fig. 4B).

In summary, AOCs represent a promising therapeutic modality for targeted delivery of various classes of ONs. However, key challenges remain, including limited understanding of intracellular trafficking, inefficient endosomal escape, complex PK/PD relationships, and insufficient tools to monitor intact AOC behavior *in vivo*. Addressing these limitations through systematic and well-controlled studies will be essential to fully realize the clinical potential of AOCs and advance their application in precision medicine.

## Supplementary Material

tbag022_Supplementary_Data

## Data Availability

No data was involved in the writing of the manuscript. For the information on the references and figures, the corresponding authors should be contacted.

## Animal research statement

No animal experiments were conducted.

## List of abbreviations

2′-F: 2′-fluoride
2′-MOE: 2′-methoxyethyl
2′-OMe: 2′-*O*-methyl
AD: Alzheimer's disease
ADA: anti-drug antibody
ADC: antibody-drug conjugate
A(D+O)C: antibody-drug-oligonucleotide conjugate
ALL: acute lymphoblastic leukemia
ALS: amyotrophic lateral sclerosis
AOC: antibody-oligonucleotide conjugate
ASO: antisense oligonucleotide
BBB: blood brain barrier
BCN: bicyclononyne
caRNA: chromatin-associated RNA
circRNA: circular RNA
CNS: central nervous system
cEt: constrained ethyl
DAR: drug to antibody ratio
DBCO: dibenzocyclooctyne
DM1: Myotonic Dystrophy Type 1
DMD: Duchenne Muscular Dystrophy
Dmpk: myotonic dystrophy protein kinase
DOPE: dioleoylphosphatidylethanolamine
EGFR: Epidermal Growth Factor Receptor
FSHD: facioscapulohumeral muscular dystrophy
GBM: glioblastoma
HER2: Human Epidermal Growth Factor Receptor 2
ICB: immune-checkpoint blockade
KRAS: Kirsten rat sarcoma viral oncogene homolog
LBPA: lysobisphosphatidic acid
LNA: locked nucleic acid
lncRNA: long noncoding RNA
mAb: monoclonal antibody
MALAT1: metastasis associated lung adenocarcinoma transcript 1
MAPT: Microtubule-Associated Protein Tau
miRNA: microRNA
MSD: multispecific drug
Mstn: myostatin
MTGase: microbial transglutaminase
MXD3: MYC-associated factor X dimerization protein 3
NHS: N-hydroxysuccinimide ester
ON: oligonucleotide
OTV: oligonucleotide transport vehicle
PD: pharmacodynamics
PEG: polyethylene glycol
PEI: polyethyleneimine
PK: pharmacokinetics
PMO: Phosphorodiamidate Morpholino Oligonucleotide
RA: rheumatoid arthritis
RGD: rare genetic disorder
RISC: RNA-induced silencing complex
SAR: structure activity relationship
saRNA: small activating RNA
scFv: single-chain fragment variable
siRNA: small interference RNA
SMCC: N-succinimidyl 4-(N-maleimidomethyl)cyclohexane-1-carboxylate
snRNA: small nuclear RNA
snoRNA: small nucleolar RNA
TCO: trans-cyclooctene
TF: transcription factor
TfR1: transferrin receptor 1
TRAAC: Toll-like Receptor Agonist Antibody Conjugate
TREM2: triggering receptor expressed on myeloid cells 2
